# The EMERALD (Enabling Mobilization, Empowerment, Risk Reduction, and Lasting Dignity) Study: Protocol for the Design, Implementation, and Evaluation of a Community-Based Combination HIV Prevention Intervention for Female Sex Workers in Baltimore, Maryland

**DOI:** 10.2196/23412

**Published:** 2021-04-16

**Authors:** Bradley E Silberzahn, Catherine A Tomko, Emily Clouse, Katherine Haney, Sean T Allen, Noya Galai, Katherine H A Footer, Susan G Sherman

**Affiliations:** 1 Department of Sociology The University of Texas at Austin Austin, TX United States; 2 Department of Health, Behavior & Society Johns Hopkins Bloomberg School of Public Health Baltimore, MD United States; 3 Department of Epidemiology Johns Hopkins Bloomberg School of Public Health Baltimore, MD United States

**Keywords:** sex work, female sex worker, recruitment, retention, sexually transmitted infection, human immunodeficiency virus, intervention, community cohesion, protocol design

## Abstract

**Background:**

Cisgender female sex workers (FSWs) experience high rates of HIV and sexually transmitted infections (STIs), including chlamydia and gonorrhea. Community empowerment–based responses to the risk environment of FSWs have been associated with significant reductions in HIV and STI risk and associated risk behaviors; however, evaluations of US-based interventions targeting FSWs are limited.

**Objective:**

The objective of this study is to describe the design, implementation, and planned evaluation strategy of an ongoing comprehensive community-level intervention in Baltimore City, Maryland, which aims to improve HIV and STI risk and cumulative incidence among FSWs. The two intervention components are the SPARC (Sex Workers Promoting Action, Risk Reduction, and Community Mobilization) drop-in center and the accompanying comprehensive mobile outreach program. The mission of SPARC is to provide low-barrier harm reduction services to FSWs, with a special focus on women who sell sex and use drugs. Services are provided through a harm reduction framework and include reproductive health and sexual health care; medication-assisted treatment; legal aid; counseling; showers, lockers, and laundry; and the distribution of harm reduction tools, including naloxone and sterile drug use supplies (eg, cookers, cotton, syringes, and pipes).

**Methods:**

The SPARC intervention is being evaluated through the EMERALD (Enabling Mobilization, Empowerment, Risk Reduction, and Lasting Dignity) study, which consists of a prospective 2-group comparative nonrandomized trial (n=385), a cross-sectional survey (n=100), and in-depth interviews assessing SPARC implementation (n=45). Participants enrolled in the nonrandomized trial completed a survey and HIV and STI testing at 4 intervals (baseline and 6, 12, and 18 months). Participants recruited from predefined areas closest to SPARC comprised the intervention group, and participants from all other areas of Baltimore were included in the control group.

**Results:**

We hypothesize that addressing structural drivers and more immediate medical needs, in combination with peer outreach, will improve the HIV and STI risk environment, leading to community empowerment, and reduce the HIV and STI cumulative incidence and behavioral risks of FSWs. Data collection is ongoing. A baseline description of the cohort is presented.

**Conclusions:**

In the United States, structural interventions aimed at reducing HIV and STIs among FSWs are scarce; to our knowledge, this is the first intervention of its kind in the United States. The results of the EMERALD study can be used to inform the development of future interventions targeting FSWs and other at-risk populations.

**Trial Registration:**

ClinicalTrials.gov NCT04413591; https://clinicaltrials.gov/ct2/show/NCT04413591.

**International Registered Report Identifier (IRRID):**

DERR1-10.2196/23412

## Introduction

### Background

Cisgender female sex workers (FSWs), defined as cisgender (ie, assigned female at birth and identifying as female) women who exchange sex for money, drugs, or goods, have 14 times the risk of being infected with HIV compared with those who do not exchange sex [1]. Similarly, cisgender FSWs experience sexually transmitted infections (STIs) at disproportionate rates [2-4]. Infectious diseases are often occupational hazards of sex work, with criminalization exacerbating HIV risk behaviors, including unprotected sex with multiple and high-risk sex partners [5,6]. The small body of research on HIV and STIs among FSWs in the United States finds high rates of HIV and STIs, similar to some other contexts globally [1,7,8].

The behavioral HIV and STI risk factors of FSWs are positioned in a broader context of enduring sociostructural vulnerabilities (eg, poverty, stigma, violence, and housing instability) that drive chronic substance use, victimization, and poor mental health, particularly in an illicit sex work market [9-11]. These factors are independently and synergistically associated with engagement in sex work and attendant HIV and STI risk [12,13] and are consistently found to be elevated among street-based and unsanctioned venue-based FSWs (eg, exotic dance clubs) compared with brothel-based FSWs [10,14,15]. The illegal nature of sex work heightens these vulnerabilities [10,14,15]. Substance use can also play a complex role in the lives of FSWs, with high rates being reported among samples of FSWs in a variety of settings [16-18]. Substance use can exacerbate engagement in risky sex and serve as a coping mechanism [19,20]. Sexual and physical abuse also heightens vulnerability to HIV and STI infection for FSWs [11,21,22]. Furthermore, FSWs have high rates of mental health morbidities (ie, depression and anxiety), which are risk factors for and underlying determinants of addiction and HIV and STI acquisition [23,24].

In response to these complicated needs and HIV and STI drivers, structural HIV and STI prevention interventions have been developed globally to address social, political, cultural, and economic factors that shape HIV and STI transmission [25-27]. Progress toward the global target of HIV elimination would not be possible without interventions and programs aimed at reducing high-risk behaviors among marginalized groups such as FSWs [28,29]. One of the most renowned and long-standing community-based structural interventions targeting FSWs, Sonagachi, addresses the burden of HIV among FSWs in Calcutta, India, with a holistic approach, including health clinics, banking cooperatives, and social services [30-32]. Using a community empowerment approach, Sonagachi prioritizes FSWs’ involvement in the implementation of the intervention, recognizes sex work as work, and operates from an understanding of the centrality of structural drivers in facilitating and mitigating the health of FSWs.

The Joint United Nations Programme on HIV and AIDS has recognized community empowerment in sex workers as the best practice for over a decade [33]. A recent meta-analysis found that community empowerment–based responses to HIV in FSWs were consistently associated with significant reductions in HIV, gonorrhea, chlamydia, syphilis, and increased condom use [34]. Although robust research exists in international settings, research on US structural interventions for FSWs is undeveloped [13,35,36]. A recent systematic review of US-based HIV and STI prevention intervention research with FSWs found that of 19 interventions, few were rigorously evaluated and none addressed the complex sociostructural context of risk [37].

The need for US-based interventions is underscored by results from our recently completed SAPPHIRE (Sex Workers and Police Promoting Health in Risky Environments) study, a prospective observational cohort study of cisgender (n=250) and transgender (n=63) street-based FSWs in Baltimore [38-46]. Cisgender FSWs showed elevated rates of structural vulnerabilities, including housing instability, food insecurity, limited education, and criminal justice involvement, and injection and noninjection drug use, compared with similarly aged peers. Structural vulnerabilities were associated with high baseline prevalence estimates of STIs, including HIV (5%), chlamydia (10%), and gonorrhea (12%) [40], and the incidence of chlamydia and gonorrhea was 13.7% and 18.2%, respectively, over 12 months [43]. Participants also reported frequent interactions with police and a high prevalence of police, intimate partner–, and client-perpetrated violence [38,44].

**Figure 1:**
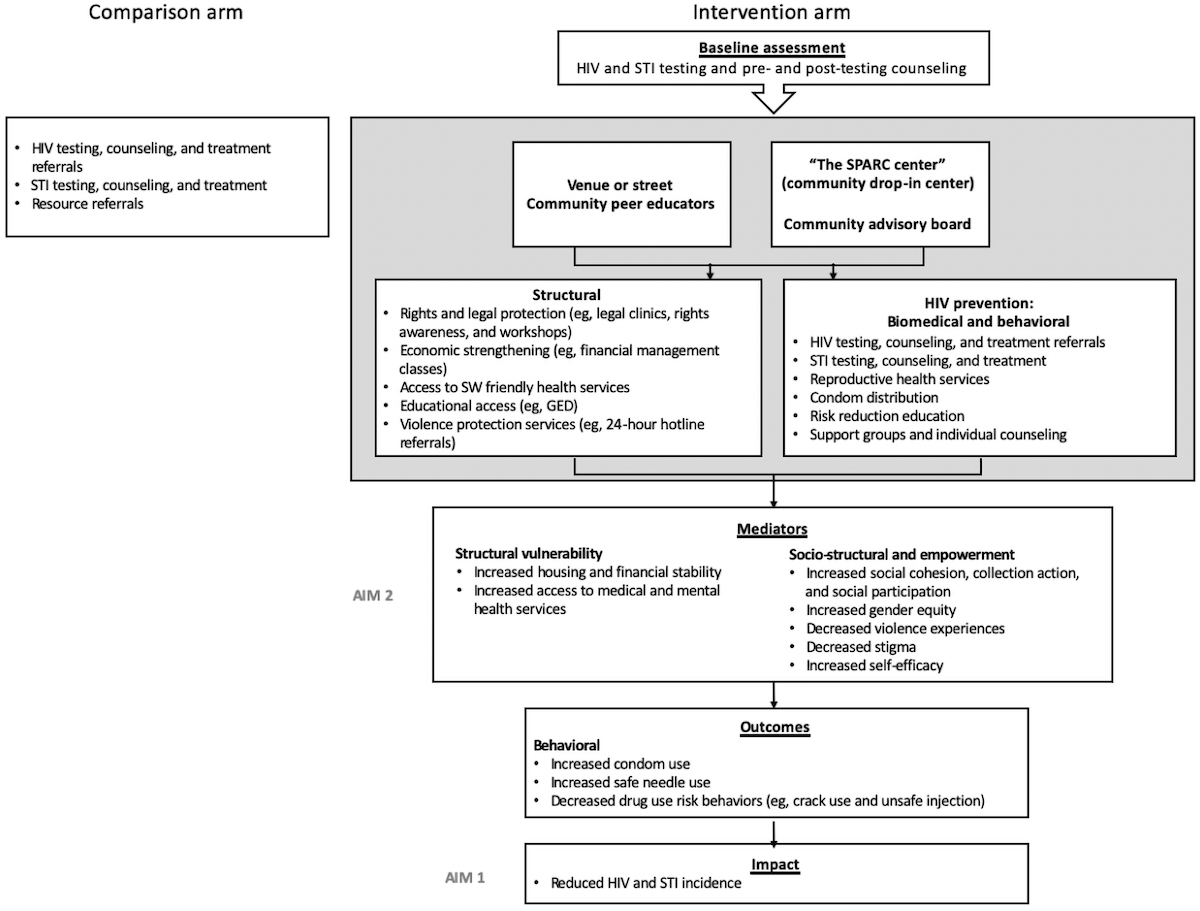
EMERALD (Enabling Mobilization, Empowerment, Risk Reduction, and Lasting Dignity) study theoretical framework. GED: general educational development; SPARC: Sex Workers Promoting Action, Risk Reduction, and Community Mobilization; STI: sexually transmitted infection; SW: sex worker.

Building on these results, we aim to develop and evaluate the effectiveness of a community-based combination HIV and STI prevention intervention in Baltimore, Maryland, to include a range of biomedical (eg, HIV and STI testing and counseling, Title X–funded reproductive health, HIV treatment, drug treatment, and primary care referrals), behavioral (eg, HIV and STI risk reduction education), and structural (eg, financial literacy, legal aid, and housing referrals) services provided through SPARC (Sex Workers Promoting Action, Risk Reduction, and Community Mobilization), a drop-in center and comprehensive outreach program. This study uses a structural determinants framework (Figure 1) to understand the complex and multifaceted nature of FSWs’ HIV risk [47]. This framework is informed by fundamental cause theory, which argues that fundamental causes (eg, poverty) have a greater impact on health than behavioral risks, and empowerment theory, which links social participation to increased social cohesion [34,48,49]. In this paper, we describe the design of the EMERALD (Enabling Mobilization, Empowerment, Risk Reduction, and Lasting Dignity) study, which evaluates the impact of the SPARC intervention. We also detail the SPARC intervention aims, components, and lessons learned during implementation. All study procedures were approved by the Johns Hopkins Bloomberg School of Public Health Institutional Review Board (JHBSPH IRB).

### Aims and Hypotheses

The EMERALD study, detailed below, aims to examine (1) the effect of intervention exposure on HIV and STI risk behaviors (eg, drug use and unprotected sex) and HIV and STI cumulative incidence over time in FSWs in the intervention group compared with those in the comparison group, (2) how sociostructural (eg, social cohesion and stigma) and structural vulnerability (eg, financial and housing stability) indicators change and are associated with the biological and behavioral outcomes over time in FSWs in the intervention group compared with those in the comparison group, (3) the role of these indicators as mediators of the intervention effect on study outcomes, and (4) the implementation of the intervention through qualitative (eg, in-depth interviews) and quantitative (eg, assessment of program fit, reach, and cost, and facilitators and barriers to utilization measures).

The SPARC center and associated outreach is the intervention being evaluated by the EMERALD study, which consists of an 18-month longitudinal cohort study comprising intervention (n=224) and control (n=161) participants, a cross-sectional survey to assess program reach (n=100), and in-depth interviews (n=45) to assess SPARC implementation. We hypothesize that addressing structural drivers and more immediate medical needs, in combination with peer outreach, will lead to community empowerment and reduce FSWs’ HIV and STI incidence and behavioral risks.

## Methods

### Intervention: The SPARC Center and Outreach Program

**Figure 2:**
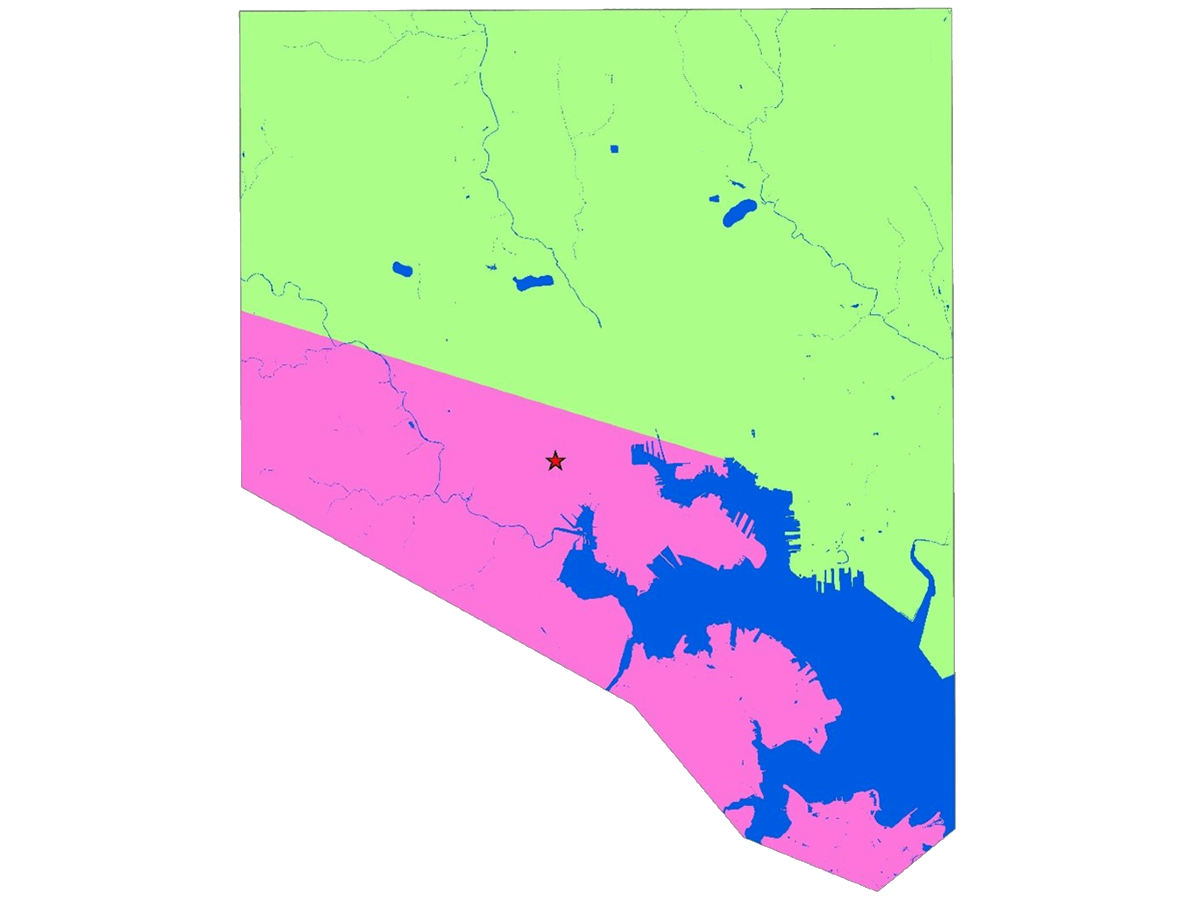
Map of EMERALD (Enabling Mobilization, Empowerment, Risk Reduction, and Lasting Dignity) intervention and control areas. Red star=SPARC (Sex Workers Promoting Action, Risk Reduction, and Community Mobilization) center, blue=water, pink=intervention area, and green=control area.

The 2 intervention components are the SPARC drop-in center and the accompanying comprehensive outreach program, both of which target FSWs in the intervention area in south and southwest Baltimore (Figure 2). SPARC opened in November 2017, and the outreach program began in earnest the following fall. The mission of SPARC is to provide low-barrier harm reduction services to at-risk nonmen, with a special focus on women who sell sex and use drugs. SPARC addresses clients’ needs through nonjudgmental, convenient, safe, and nonstigmatizing interactions, reducing the need for outside referrals and increasing the likelihood of continued engagement in care and service utilization. All services are provided through a harm reduction framework, centering the lived experiences of those served, while addressing the structural and socioeconomic constraints that often limit their choices and options. Ultimately, SPARC’s overarching goal is to foster a sense of community and stimulate empowerment by creating a safe physical space in which women can connect and develop social cohesion and a sense of collectivism.

SPARC’s day-to-day operations are bolstered by key collaborations with community providers who offer services at SPARC, enabling these organizations to reach a population they otherwise do not easily access. The Baltimore City Health Department funds 2 nurse practitioners and a medical assistant to run a weekly reproductive health clinic (eg, long-acting reversible contraceptives, Papanicolaou testing, and intrauterine devices) and a twice-weekly sexual health clinic (eg, pre-exposure prophylaxis, HIV and Hepatitis C testing and treatment referrals, and STI testing and treatment). The Behavioral Health Leadership Initiative funds 2 providers to staff a weekly low-threshold medication-assisted treatment clinic at SPARC. Biweekly law clinics are offered through the Legal Aid of Maryland and the Maryland Volunteer Lawyers Network.

SPARC’s reach is expanded through an intensive mobile outreach program, which brings many tangible harm reduction tools and microcounseling to women while they are working and living on the street. The outreach program is deployed throughout south and southwest Baltimore at night and during the day between 3 and 4 times a week, serving 15 to 40 people per shift. Outreach staff work in teams to provide relevant harm reduction education and supplies from the window of the outreach vehicle, through drop-off supply bags, and during community events.

All SPARC and outreach staff receive extensive training as a part of onboarding and continuing education. Training sessions are conducted by the study staff as well as external experts and cover topics such as trauma-informed care, harm reduction principles and practice, interpersonal and systemic violence, safer drug use (eg, safer injection), naloxone administration, gender and sexual identity, and de-escalation and staff safety practices. Understanding that trauma often serves as a direct barrier to care, much of the continuing education at SPARC centers around trauma-informed care and supporting the complex needs of vulnerable individuals.

To ensure equal treatment of all guests, the SPARC center and outreach staff are blind to EMERALD study participation. Limited guest information is collected at intake, primarily their name and birthdate, sex work and drug use history, and service needs. This information allows the team to track service utilization among all women to inform programming and determine whether the intervention is reaching the target population. SPARC guests are asked to sign a consent form indicating that they agree to allow the research team to access their SPARC data. Unique identifiers will be linked between EMERALD and SPARC to determine the frequency of SPARC center interactions among EMERALD participants at the conclusion of all EMERALD data collection. Owing to the quick pace and casual nature of outreach, no identifying information is collected on outreach shifts.

The SPARC program contributes to the innovation of a service model that incorporates health, social, legal, and basic needs (eg, emergency food, shower, laundry, and space to relax) services in a convenient space, paired with a comprehensive outreach program that brings services, supplies, and information to the community, increasing the likelihood of engagement in care and continued service utilization.

### The EMERALD Study

#### Study Design

The primary component of the EMERALD study, which evaluates the SPARC center and associated outreach, is a prospective 2-group comparative nonrandomized trial. Participants were recruited into the control and intervention arms. The intervention area is the south and southwest portion of Baltimore City because of high concentrations of street-based FSWs and a dearth of tailored FSW services, indicated by pink shading (Figure 2). The control group participants were recruited primarily from the southeast and northwest Baltimore. The SPARC center is located within the intervention area, and outreach is conducted in areas with potential sex work activity throughout the intervention area.

**Figure 3:**
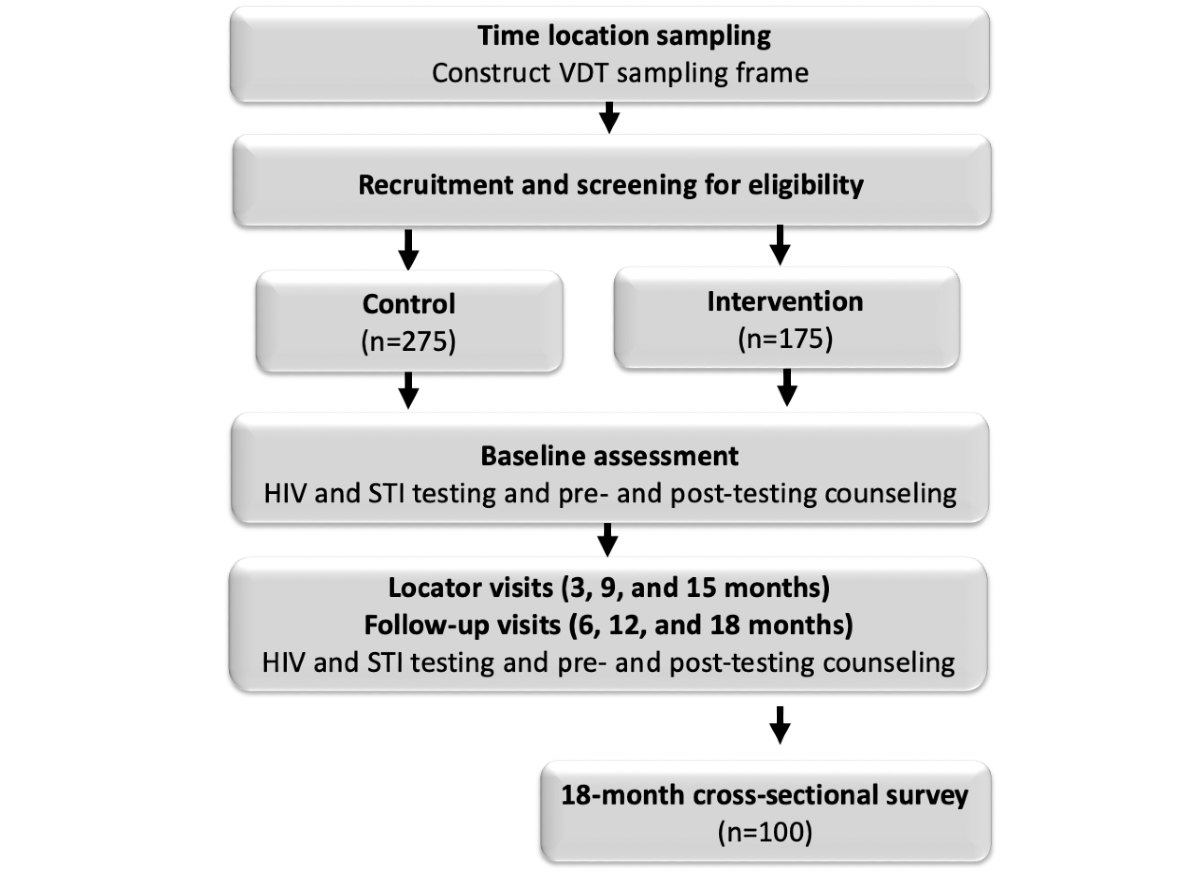
EMERALD (Enabling Mobilization, Empowerment, Risk Reduction, and Lasting Dignity) study flow. STI: sexually transmitted infection; VDT: venue-date-time.

The study diagram (Figure 3) illustrates participant progress through the study stages, sample size, and follow-up schedule. Participants completed a survey, HIV testing, and STI testing for chlamydia and gonorrhea during study visits at baseline and at 6, 12, and 18 months. In-depth interviews began at the conclusion of the EMERALD cohort and are currently being conducted with intervention area participants, SPARC staff, and outreach workers. In addition, 100 cross-sectional surveys are being collected in the intervention area to assess program reach in the community beyond the EMERALD cohort.

#### Cohort Study Target Population and Sample

Cohort participants were FSWs aged ≥18 years who live in Baltimore City; self-reported that they had traded sex for money, goods, or drugs 3 or more times in the past 3 months; and were not currently enrolled in our previous cohort study, the SAPPHIRE study [40]. Although transgender female sex workers (TFSWs) who reported being assigned male at birth were encouraged to visit the SPARC center and engage with peer outreach workers, they were excluded from the EMERALD study cohort because of a lack of comparable recruitment locations between the intervention and control areas.

Participants were assigned to a study arm based on the geographic location in which they were recruited. The initial target sample size was 350 women based on power calculations to observe a change in the intervention compared with the control group over the 3 follow-up visits. The assumptions used for the calculation were (1) n=200 in the intervention group and n=150 in the comparison group, (2) lost-to-follow-up (LTFU) rate of 10% to 20% in the intervention (effective n=160-180) and LTFU of 20% to 25% in the comparison group (effective n of 120-112), (3) intraperson correlation (Rho)=0.2-0.4, (4) varying levels of baseline risk behavior rate, and (5) a 2-sided α=.05. Thus, assuming effective sample sizes of n=160 in the intervention and n=112 in the comparison groups, the power is 83% to detect an odds ratio of 0.50, assuming a 20% prevalence rate of a risk behavior (eg, drug use) in the control group and an intraperson correlation of Rho=0.2. For the cumulative incidence outcome, with an average N contributing to the analysis of 170 in the intervention and 120 in the control groups, and assuming annual incidence rate of 6%, the power is 80% to detect a rate ratio of 0.28.

The enrollment target was increased to 450 midway through data collection after power calculations were reassessed based on actual retention rates within the sample, which were closer to 65%. The LTFU rate was higher than anticipated based on previous studies [41], likely because of high incarceration rates, the mobility of the population, and the length of time between study visits (6 months). Although we aimed for a sample of 450 participants, recruitment was stopped at 385 because of reaching saturation in the recruitment areas and repeatedly encountering participants attempting to reenroll in the study.

#### Cohort Recruitment

EMERALD recruitment began 2 months before the opening of the SPARC center and took place from September 2017 to January 2019. The recruitment strategy for the EMERALD cohort was informed by SAPPHIRE, an earlier prospective cohort study of FSWs in Baltimore, conducted by this study team, which used targeted sampling [50]. Given that recruitment for SAPPHIRE concluded approximately 9 months before the launch of the EMERALD study, we conducted a series of geospatial analyses of possible sex work activity indicators using publicly available data [51] (eg, prostitution charge data and 911 call center reports of suspected prostitution) to understand possible shifts in the geotemporal distribution of sex work throughout Baltimore City (as compared with where we previously recruited FSWs in SAPPHIRE).

In total, the sampling frame for EMERALD consisted of 10 small geographic areas with high concentrations of sex work activity. Among them, 6 were located in the intervention area and 4 in the control area. Compared with our previous targeted sampling recruitment strategy among FSWs in Baltimore City [50], the time and day components of the sampling frame were selected based on analyses of time signatures associated with relevant secondary data in each location with high concentrations of sex work activity and supplemented with additional insights gleaned from SAPPHIRE. Before launching recruitment, staff conducted windshield tours in each location to confirm the presence of sex work activity.

A recreational vehicle (RV) retrofitted with 2 private interview areas and a bathroom for sample collection was driven to the field locations in the intervention and control areas 3 to 5 days per week depending on the interview volume. Each shift lasted approximately 4 hours at the recruitment site and was staffed by 1 field supervisor and 2 interviewers. A community advisory board made up of current and former sex workers provided input on all data collection strategies and instruments.

Potential participants were discreetly approached by staff who provided a brief description of the study. Due to the sensitive nature of sex work, drug use, and HIV, study staff were trained to refer to the study as a women’s health study when communicating with potential participants or community members. Women who were interested in participating were offered screening in a private area on the study RV. FSWs who were eligible and interested in participating in the study were asked to provide written informed consent for all study procedures using an electronic consent form. Following the consent process, participants completed a standard locator form, which included mobile and home phone numbers, addresses, social media usernames, places frequented by the participant, and contact information of family or friends. This locator information is collected and updated at every visit to increase the likelihood of successful retention for future study visits [41].

After completing the locator form, participants received HIV pretest counseling from trained staff and were tested for HIV using the OraQuick *ADVANCE* Rapid HIV-1/2 Antibody Test, which detects HIV-1 and HIV-2 antibodies in saliva in 20 minutes. While waiting for the results of their HIV test, participants completed a 45- to 60-minute audio-enhanced computer-assisted self-interviewing (A-CASI) survey, with staff on hand to provide assistance. The use of A-CASI provided uniformity of delivery and afforded greater privacy and confidentiality than an interviewer-administered survey, considering the sensitive nature of some survey questions. The baseline survey assessed sociodemographic characteristics, arrest and prison history, sex work history, frequency and type of police encounters, drug and sexual-risk behaviors, overdose and drug treatment history, general health history, experiences of sexual and physical violence, FSW social cohesion, stigma, empowerment, health service utilization, and a brief screening for posttraumatic stress disorder and depression symptoms (Table 1).

**Table 1:** Variables reflected in the conceptual framework and collected as part of the EMERALD (Enabling Mobilization, Empowerment, Risk Reduction, and Lasting Dignity) study of female sex workers in Baltimore, Maryland.

Variable		Example question
**Outcomes and impact**		
	HIV diagnosis	N/A^a^
	Sexually transmitted infection diagnosis	N/A
	Substance use	Have you (injected, smoked, swallowed, or snorted—asked separately) any of the following drugs? Powdered cocaine Crack cocaine Heroin Fentanyl Buprenorphine or suboxone Prescription pain relievers Sedatives or tranquilizers Stimulants
	Condom use	In the last week, how often did you use condoms when having (vaginal or anal) sex with (clients or intimate partner)? Always Most of the time Sometimes Rarely Never Did not have (vaginal or anal) sex
	Safe drug use practices	In the last 6 months, did you use any of the following items that you know have been used by someone else? Syringes or needles Cookers Cotton None of these
**Confounders**		
	**Housing stability**	
		Have you been homeless in the past 6 months?
		Where do you currently stay? Place that you own Place that you rent Family member’s place Shelter Transitional housing program Hotel or motel Streets, park, car, or abandoned building
	**Financial stability**	
		In the past 6 months, did you depend on anyone financially? This includes for food or a place to stay.
		Do you currently owe money to anyone, including a person, company, or a bank?
	**Access to physical and mental health services**	
		Was there a time in the past 6 months when you wanted or needed to see a doctor or health care provider (other than addiction services) but could not?
	**Social cohesion**	
		You can count on other people who sell sex if you need to borrow money; 4-point Likert scale (strongly disagree to strongly agree)
		You look out for new girls when they start selling sex on the street; 4-point Likert scale (strongly disagree to strongly agree)
	**Collective action and social participation**	
		Do you participate in any of the following: Church Clubs Cultural activities Community organizations
	**Gender equity**	
		Men’s opinions are more important than women’s in making important decisions in a primary relationship; 4-point Likert scale (strongly disagree to strongly agree)
		If a man wants to have sex in a primary relationship and a woman does not, she should have sex to please him; 4-point Likert scale (strongly disagree to strongly agree)
	**Violent experiences [52]**	
		Thinking about your (clients or intimate partners): Have you been hit, punched, slapped, or otherwise physically hurt by them? Have they removed a condom during sex after agreeing to use one?
	**Sex work stigma [53]**	
		There are times you feel ashamed of selling sex; 4-point Likert scale (totally disagree to total agree)
		People’s attitudes about selling sex make you feel worse about yourself; 4-point Likert scale (totally disagree to total agree)
	**Empowerment [54]**	
		I have little control over the things that happen to me; 5-point Likert scale (totally disagree to total agree)
		There is little I can do to change many of the important things in my life; 5-point Likert scale (totally disagree to total agree)

^a^N/A: not applicable.

On completion of the survey, participants were asked to self-collect vaginal swabs with Aptima vaginal swabs (Hologic Inc) using the private RV bathroom. The samples were sent to the Baltimore City Health Department lab, which conducted nucleic acid amplification testing for gonorrhea and chlamydia. Once samples were collected, the participants received the results of their HIV test and posttest counseling. A 2-week appointment was scheduled to receive the STI test results.

Participants were given a US $70 prepaid Visa debit card as compensation for their time and offered relevant referrals to health and social services. Participants recruited from the intervention area were encouraged to visit SPARC and offered hygiene products such as sanitizer wipes and lip balm with SPARC branding, including the address and phone number of the center. Control area FSWs were offered similar EMERALD-branded items and provided neighborhood-specific referrals if requested but were not encouraged to visit SPARC.

#### Cohort Retention Methods

Follow-up visits for EMERALD occurred at 6, 12, and 18 months after the initial baseline interview. Participants received US $40 prepaid Visa debit cards for completing each follow-up visit. Participants were eligible to complete each follow-up visit 1 month before their actual interview date until 1 month after, for a total eligibility window of 2 months. Those who failed to complete an interview within the allotted eligibility window were considered lost to follow-up. Participants who missed a visit could still complete their next scheduled visit and were not withdrawn from the study.

Retention protocols were modeled after strategies used in SAPPHIRE [50] and are more fully described in a study by Silberzahn et al [41]. Briefly, retention strategies consisted of the aforementioned locator forms (eg, phone numbers, addresses, and social media accounts); promotional materials (eg, hand sanitizer, wipes, lip balm, and silicon bracelets) branded with the study logo and phone number; monitoring public information databases to determine if participants were incarcerated, had moved, or were deceased; and outreach in the form of phone calls, social media messaging, home visits, and repeated visits to recruitment areas in both the study RV and a study sedan, which was used for follow-up. The study team also relied on tracking teams of 2 study staff who were assigned participants to focus on with 1 month of eligibility remaining.

Building on the strategies used in SAPPHIRE, the EMERALD cohort retention protocols were enhanced using several additional strategies. A password-protected database was developed to monitor follow-up and participant progress during the study visits. Using this database, study management could easily determine current and projected follow-up rates, the number of participants due for a study visit (6, 12, and 18 months), and all previous contact attempts used to locate a participant. Field staff documented how participants were located (eg, phone calls and home visits) and time spent on van shifts and tracking, which allowed study management to determine adequate staffing and consider the best follow-up strategies. For example, study staff could monitor the number of difficult-to-reach participants due for follow-up surveys in specific study areas and direct tracking teams to these locations to increase the probability of chance encounters with participants. Additionally, check-in locator visits were conducted in between formal study visits at 3, 9, and 15 months to touch base with participants and inquire about any changes to contact information on file (eg, phone numbers, addresses, family and friends). Finally, although SPARC staff were prohibited from discussing the EMERALD study with clients, participants could obtain the EMERALD study phone number from SPARC center administrative staff at their request and use the SPARC office phone to contact the EMERALD study staff, determine follow-up eligibility, and schedule follow-up interviews.

#### In-Depth Interviews and Cross-sectional Surveys

In-depth interviews and cross-sectional surveys began at the conclusion of the 18-month EMERALD cohort. In total, thirty 45- to 60-minute in-depth interviews will be conducted with intervention cohort members who did (n=15) and did not (n=15) visit the SPARC center, and 15 interviews will be completed with SPARC center staff and outreach workers. The EMERALD cohort participants receive a US $25 prepaid Visa debit card for completing the interview; however, SPARC staff are not paid for participation. Interviews with intervention area cohort participants assess program fit, facilitators, and barriers to access of SPARC center services. Respondents are asked about the convenience of hours of operation, center location, staff, and services. Participants are also asked what they liked or disliked about the program and recommendations for additional services. Interviews with SPARC staff examine facilitators and barriers to the success of SPARC and how to improve services and reach.

We are further assessing the intervention’s reach (eg, peer educator contact) through a cross-sectional survey at 18 months among cisgender women aged ≥18 years (n=100) recruited from the intervention areas. Surveys are interviewer administered; take approximately 10 minutes to complete; and consist of questions regarding sex work history, knowledge, and utilization of SPARC services. Participants receive a US $5 prepaid Visa card for completing the cross-sectional survey.

#### Study Monitoring

The EMERALD study team has several key roles: the principal investigator and co-investigators, study director, field supervisor, field staff, and data manager. Investigators and the study director are responsible for protocol development and the oversight of the study progress. Field staff are responsible for data collection, and the data manager monitors and analyzes all study data to ensure that the data are of the highest quality. EMERALD investigators and the research team meet weekly to obtain feedback from field staff regarding study protocols and to discuss study progress. On the basis of staff feedback, data collection protocols are updated and subsequently sent to the JHBSPH IRB for approval. Any adverse events are reported to the JHBSPH IRB within 10 days, as required. Annual progress reports are also submitted to the study sponsor and to the JHBSPH IRB, documenting study progress. Finally, an independent and external data safety monitoring board comprising individuals with relevant expertise provides oversight and monitors study progress.

### Statistical Analysis

The principal analytic approach will center on generalized linear models for the individual data, with variance components that reflect the potential correlations among FSWs recruited from the same area and between repeated measurements from the same individual at baseline and follow-up visits [55,56]. The main covariate of interest is the intervention indicator. To address aim 1, we consider illicit drug use and irregular condom use as the primary behavioral endpoints.

The effect of the intervention will be assessed with a random effect hierarchical logistic model, with the intervention indicator, time, and their interaction as the main explanatory variables. To adjust for possible imbalances between the 2 areas, we will adjust for baseline confounding factors, including age, length of sex work, and number of sexual partners. The intervention effect will be assessed by comparing time trends in the intervention group with the time trend in the comparison group, as captured by the interaction term. The model will be expanded to include additional demographic covariates and time-dependent indicators of events such as arrest, homelessness, use of health services, or exposure to other prevention activities that are unrelated to the study. Aim 1 also seeks to evaluate the association between the degree of exposure to the intervention and observed behavioral changes. We will explore models that include as covariates participation or exposure to specific intervention components (ie, receiving medical care, drug treatment referral, and legal aid). On the basis of the literature, an 18-month follow-up should be sufficient to document meaningful changes in study outcomes, given the extent of the intervention [57].

For the primary biological endpoint of HIV and STI incidence, we will initially estimate cumulative incidence rates in both the intervention and comparison participants as a binary outcome and construct confidence intervals, accounting for the correlation structure because of nesting in geographic recruitment areas. The intervention’s effect will be assessed using an unadjusted random effect logistic model, with the intervention indicator as the only explanatory variable. The model will be expanded to include baseline covariates, such as demographic characteristics, history of sex work, and baseline risk behaviors (ie, condom use). A more detailed analysis will employ person-time methods with Poisson regression models to allow for time-updated covariates measured over time. Those lost to follow-up will contribute time-at-risk until the time of their last visit.

Finally, to evaluate the reach of the intervention, we will estimate SPARC involvement of FSWs in the independent cross-sectional survey at the 18-month follow-up period. We will also compare this sample with the FSW cohort at 18 months to assess their comparability or differences regarding demographic and risk profiles using logistic regression with group membership as the outcome variable.

To address aim 2, we will first describe changes over time in various sociostructural (ie, social cohesion and stigma) and structural vulnerability (eg, housing stability) indicators. We will then contrast these trends between the intervention and comparison groups. Social cohesion, social participation, and collective action will be analyzed as aggregate measures, the form of which will be determined by exploratory analysis. Specific models for formal statistical comparisons will be developed based on exploratory analysis, identifying both the distributional properties of the indicators and the shape of changes over time. To assess the association of these changes with the study’s primary endpoints, we will develop random effects logistic models, where the changes in structural indicators at each visit compared with baseline will be the main explanatory factors, in addition to the intervention indicator. The random effects will reflect the nesting of observations within the recruitment area and person over time.

To address aim 3, we will carry out mediation analyses evaluating the contribution of intermediate endpoints that were targeted by the intervention on the primary study outcomes. These include sociostructural factors (eg, stigma and social cohesion) and behavioral outcomes (illicit drug use and irregular condom use). We will have limited power to perform a definitive mediation analysis, but we plan to run detailed descriptive analyses and develop initial models to evaluate potential mediators that could be later validated in a larger study. The analysis will be based on recent developments in mediation analysis that extend earlier results to outcomes that are binary, such as irregular condom use. Specifically, the mediation formula proposed by Pearl [58] and the general framework for causal mediation analysis by Imai et al [59] will be adopted. Full data analysis and power calculation details are available from the senior author upon reasonable request.

To address aim 4, interviews with staff, FSWs, and peer educators will be entered and managed separately using ATLAS.ti software (Scientific Software Development GmbH). Coding will reduce the data to manageable units of information that cover broad and general categories. Themes that emerge from the data will be analyzed using a grounded theory approach [60,61]. A total of 2 coders will conduct open coding on 3 transcripts to develop initial coding schemes. After discussion and development of a combined draft scheme, 2 additional interviews will be coded to inform the final coding scheme. Discrepancies will be discussed with the interviewers and the primary investigator. Through weekly analysis meetings, a team approach to data analysis will be employed, whereby different researchers provide feedback on emerging interpretations and check emerging categories against the raw data. An audit trail will ensure the trustworthiness of findings, gather input from multiple perspectives, and enhance reliability [62].

## Results

### Recruitment and Retention

At the conclusion of EMERALD cohort recruitment, 93.9% (596/635) of the women who were approached agreed to be screened for eligibility. Reasons for ineligibility of women included: no history of selling sex (n=43), not selling sex 3 or more times in the past 3 months (n=48), not cisgender (n=2), currently enrolled in the SAPPHIRE study (n=15), currently enrolled in EMERALD (ie, enrolled participant rescreening; n=6), and inability to complete study procedures (eg, too intoxicated to give consent, too tired, and not available for enough time; n=12). A total of 470 women who were screened were eligible, provided written informed consent, and completed all baseline study procedures. After the baseline screening, 85 women were withdrawn from the study, and their data were removed from the data set. The reasons for withdrawal included already enrolled or duplicate (n=57), protocol issues such as incomplete data (n=13), participant chose to withdraw (n=2), or other reasons (n=13). When duplicates were discovered, only the first survey was included in the data set. For a participant to be removed for incomplete data, substantial portions of the survey needed to be incomplete, such as skipping a majority of sections or choosing “refused to answer” or “don’t know” for a majority of survey answers. This resulted in a final analytical sample of 385.

### Cohort Participant Characteristics

The final sample of the cohort (n=385) had a mean age of 37 years (SD 9.3 years), 56.6% (218/385) of participants were White, 70.9% (273/385) had attained less than a college education, and approximately two thirds of the sample (257/385, 66.8%) had a recent history of homelessness (Table 2). There were significant differences in racial categories between the intervention and control areas (*P*=.04). Women reported an average of 13 years (SD 9.5 years) in sex work. The prevalence of current substance use was high: 57.9% (223/385) injected any drug, 80.3% used heroin via any method, and 86.8% (334/385) used powdered or crack cocaine in the past 6 months. Almost half of the sample (174/385, 45.2%) reported a recent condomless sex with clients. HIV prevalence in the full sample was 5.2% (20/385), although only 3 women received new HIV diagnoses from study-related testing. Baseline gonorrhea and chlamydia prevalence were high at 15.8% (59/373) and 18.2% (68/374), respectively. There were no significant differences between the control and intervention groups in the key study outcomes at baseline.

**Table 2:** Baseline characteristics of a sample of female sex workers in Baltimore, Maryland, recruited to the EMERALD (Enabling Mobilization, Empowerment, Risk Reduction, and Lasting Dignity) study (n=385).

Variables						Total (n=385)			Intervention (n=224)			Control (n=161)			*P* value	
**Personal background**																
	Age (years), mean (SD)				37.0 (9.3)			37.6 (9.4)			36.3 (9.0)			.17		
	**Race**															.04
		White, n (%)		218 (56.6)			139 (62.1)			79 (49.1)						
		Black, n (%)		139 (36.1)			72 (32.1)			67 (41.6)						
		Other race, n (%)		28 (7.3)			13 (5.8)			15 (9.3)						
	**Education**															.43
		Less than high school graduate, n (%)		177 (46.0)			97 (43.3)			80 (49.7)						
		High school graduate or General Educational Development, n (%)		96 (24.9)			60 (26.8)			36 (22.4)						
		Some college or greater, n (%)		112 (29.1)			67 (29.9)			45 (28.0)						
	**Sexual orientation^a^**															.36
		Heterosexual or straight, n (%)		260 (67.7)			156 (70.0)			104 (64.6)						
		Lesbian, queer, or same gender loving, n (%)		24 (6.3)			11 (4.9)			13 (8.1)						
		Bisexual, n (%)		100 (26.0)			56 (25.1)			44 (27.3)						
	Homeless in the past 6 months, n (%)				257 (66.8)			151 (67.4)			106 (65.8)			.75		
**Sex work history**																
	Time in sex work (years), mean (SD)^b^				13.2 (9.5)			13.3 (10.0)			13.1 (8.9)			.80		
	**Found clients in the past 6 months**															
		**Street**													.97	
			Street-based only, n (%)	124 (32.2)			72 (32.1)			52 (32.3)						
			Street or other methods, n (%)	260 (67.7)			152 (67.9)			108 (67.5)						
		Web-based or mobile app^b^, n (%)		95 (36.5)			44 (29.0)			51 (47.2)			.001			
		Bar, club, or massage parlor^b^, n (%)		161 (61.9)			88 (57.9)			73 (67.6)			.17			
		Referrals from others (sex workers, pimps or managers, or intimate partners)^b^, n (%)		152 (58.2)			90 (59.2)			62 (56.9)			.71			
**Prevalence of HIV and sexually transmitted infections and risk behaviors, n (%)**																
	Injected any drug in the past 6 months				223 (57.9)			129 (57.6)			94 (58.4)			.88		
	Used heroin in the past 6 months				309 (80.3)			178 (79.5)			131 (81.4)			.64		
	Used powdered or crack cocaine in the past 6 months				334 (86.8)			195 (87.1)			139 (86.3)			.84		
	**Reused injection equipment in the past 6 months**															
		Syringes or needles^c^		68 (28.5)			42 (29.6)			26 (26.8)			.64			
		Cookers^c^		106 (44.5)			62 (43.7)			44 (45.4)			.80			
		Cotton^c^		77 (32.2)			44 (31.0)			33 (34.0)			.62			
	Condomless sex with clients in the past week				174 (45.2)			98 (43.8)			76 (47.2)			.50		
	Positive HIV rapid test^a^				20 (5.2)			12 (5.4)			8 (5.0)			.85		
	New HIV-positive diagnoses^a^				3 (0.8)			1 (0.5)			2 (1.2)			N/A^d^		
	Positive gonorrhea^e^				59 (15.7)			30 (13.7)			29 (18.6)			.20		
	Positive chlamydia^e^				68 (18.1)			33 (15.1)			35 (22.2)			.08		

^a^<1% of data missing.

^b^Of 260 women who sold sex on other locations than street.

^c^Of 239 women who injected drugs.

^d^N/A: not applicable.

^e^<3% of data missing.

Currently, all participants have moved through their 6-month, 12-month, and final 18-month visit windows. The mobile data collection strategy for EMERALD resulted in the enrollment of 385 FSWs into the cohort portion of the study. We have begun the final cross-sectional surveys, with 4% completed. A total of 11 in-depth interviews have been completed with staff and 16 with clients.

## Discussion

### Principal Findings

In the United States, structural interventions aimed at reducing HIV and STIs among FSWs are scarce; to our knowledge, this is the first intervention of its kind in the United States. Innovative and adaptable approaches for linking FSWs to health and social services are required. The findings from the EMERALD evaluation will prove useful for tailoring HIV prevention for FSWs and creating a sustainable community-based intervention. Planned dissemination of study findings includes manuscript publications, conference presentations, and community dissemination (eg, infosheets and community meetings).

The SPARC center has had great success building relationships with FSWs and engaging them in clinical and social services. Much of this success is attributed to the synchronization of street- and van-based outreach efforts that focus on harm reduction education, supply distribution, and referrals with a drop-in center space that has on-site health, social, and legal services tailored to the needs of FSWs and women who use drugs. SPARC has the capacity to provide these wide-ranging services through partnerships with local organizations interested in connecting to the population SPARC serves. SPARC provides space in the drop-in center, and partner organizations use the space to offer clinic hours to potential clients who they might not otherwise be able to access. SPARC clients have greater access to services, and the organizations are able to reach a wider segment of the population they seek to serve.

### Limitations

Despite successful implementation and the novelty of this intervention in a US setting, we encountered several challenges throughout this study. Although TFSWs experience disproportionately high rates of victimization and are underrepresented in research, a lack of comparable recruitment locations in the intervention and control areas prohibited the inclusion of TFSWs in our study [63,64]. In addition, the onset of the COVID-19 pandemic in March 2020 required adaptations to service provision and data collection. In-person services at the SPARC center were stopped on March 17, 2020. However, because the framework was already in place, the SPARC team was able to quickly pivot to an all-outreach service model when the COVID-19 pandemic forced the closure of the drop-in center.

Regarding the EMERALD evaluation, data collection for the cross-sectional survey was paused at the onset of the COVID-19 pandemic, and data collection was shifted to a telephone format for the remaining 18-month surveys. As a result, we were unable to conduct HIV and STI testing for 27 participants. We plan to reopen in-person services at the SPARC center, resume the 96 in-person cross-sectional surveys, and complete the 19 remaining in-depth interviews when data collection with human subjects is deemed safe and appropriate.

## Abbreviations

A-CASI: audio-enhanced computer-assisted self-interviewing
EMERALD: Enabling Mobilization, Empowerment, Risk Reduction, and Lasting Dignity
FSW: female sex worker
JHBSPH IRB: Johns Hopkins Bloomberg School of Public Health Institutional Review Board
LTFU: lost-to-follow-up
RV: recreational vehicle
SAPPHIRE: Sex Workers and Police Promoting Health in Risky Environments
SPARC: Sex Workers Promoting Action, Risk Reduction, and Community Mobilization
STI: sexually transmitted infection
TFSW: transgender female sex worker
